# Convalescent Homes in the Eastern Counties

**Published:** 1888-02-18

**Authors:** 


### Convalescent Homes in the Eastern Counties.

By Our Roving Correspondent.

(*Continued from p.* 338.)

The three children I have referred to as being indoors were amusing themselves in the new play-room, a glass-covered apartment, which is peculiarly suited for the colder weather; for the Home now keeps open all the year round, except in March and April, when the nor'easters are about and the whitewashers and craftsmen of that sort may most conveniently be introduced. Another very pleasant room on the ground floor is the dining-room, which commands a splendid view of the German Ocean and the low cliffs that run along the shore to the north. I ought to have said which *did* command, not which commands; for the Town Council—to do justice to whose philistinism would task the eloquence of Mr. Matthew Arnold—have permitted, for a consideration, the building of a monstrosity in hideous rough yellow timber, known as a switch-back railway, which has thrust itself between the Parade and a magnificent marine prospect. To think that for years to come the idea of the sea will be associated in the minds of the town-bred inmates of the Home with an ugly noisy switch-back railway! Proceeding upstairs, I was conducted through the dormitories, clean, bright, and well appointed, with a nurse's room on each floor so constructed as practically to command all the rooms on that level. In one room the six little beds are covered with quilts on which Scripture texts have been beautifully worked by a lady friend; and in another a bed has been contributed by the parishioners of Thorpe St. Andrew, in memory of six children who died during an epidemic of measles. Would that all memorials might take so useful a form. The Home has room for a few more cots, the furnishing of which costs only £6 each.

A word or two as to the rules by way of conclusion. The usual period for which children are kept in the Home is one month, and for this term a subscriber can present one child per annum for every guinea he subscribes during the year. A donor of twenty guineas is a life governor, and is entitled to send one child each year for a month. Of course patients recovering from any contagious disease, or subject to fits, are not admitted; and a subscriber who desires that his presentee shall remain longer than a month can secure this by the payment of 7*s.* 6*d.* a-week. For the same sum paying patients are admitted, and of these some five or six have been received during the present year. These are very welcome when there is room for them, and they are treated in every respect exactly as the other children. Experience here, as elsewhere, shows that when the Home is full the rate of expenditure per head can be kept at a lower figure than when some of the cots are empty.

The other Institution which I visited was at Felixstowe, and is called the Suffolk Convalescent Home and Sea-Bathing Infirmary. It has grown old, comparatively speaking, in the good work of caring for the sick, for it is now in the nineteenth year of its useful career, having been started in 1868, with accommodation for only eight patients. It houses some sixty men and women, and its history has been one of steady but continual growth. It presents as sharp a contrast to the Children's Home at Yarmouth as could well be imagined. There are no switchback railways, or other abominations, for Felixstowe is almost innocent of roads, and the Convalescent Home is only one of a number of villas which have been dropped down here and there in picturesque confusion. Its situation could not be better than it is. The houses—there are two of them joined into one—are backed by a high cliff which effectually prevents any land breezes from attacking the inmates; and the front windows look out upon a charming bay sheltered from the worst of the storms which sweep across the North Sea. Hitherto the Home has been closed from Christmas to May, but this year it is hoped that it may remain open until March; for Mrs. Pryke, the popular matron, ridicules the commonly-received notion that the East Coast is uninhabitable in the winter, and tells the envious Londoner how, when he is miserable with the November and December fogs, she and her friends and her patients spend some very pleasant hours each day on the beach—where, by the way, the Home has a bathing tent of its own. Machines, it should be explained, have only been recently introduced by a few revolutionary spirits.

Inside the Home a very remarkable contrast is afforded by the two houses. The older building accommodates twenty-eight women and children (the latter always over five years of age), and the rooms are such as one ordinarily meets with in a decent house. That they are scrupulously clean, bright, and pleasant goes without saying. But the latest addition—the men's wing, which has thirty beds, with the usual dining-rooms, sitting-rooms, etc.—has been built especially for the Home, and so the men, besides having all the comforts which the women's side possesses, have this further advantage, that their dormitories are erected in accordance with the latest scientific *dicta*, and are, therefore, better ventilated and altgether more healthful. Mrs. Pryke is very desirous of rebuilding the old house on the same plan, and the committee would gladly undertake the work could they see their way to raising the £4,000 which would be required. The matron herself has a novel way of adding to the resources of the Institution she governs which might be followed with advantage at other seaside places. Every year, in the height of the season, there is a sale of work on the beach opposite the Home. Felixstowe makes a gala day of the occasion, and every year the proceeds have risen. Last July the sale of work realised a hundred guineas, and as the popularity and size of Felixstowe increases, so will the success of Mrs. Pryke's bazaar. I forgot to say that a system of warm sea-water baths is among the conveniences of which the Home boasts.

This Institution owes its birth to the benevolence of the people of Ipswich, but its friends are now found in all parts of the country, and its patients come from an equally wide area. Offertories are made in many of the parish churches in the county, and under the head of "collections" in the yearly balance-sheet many "concerts" are to be found. The Oddfellows and the Foresters at Ipswich hold a joint *fête* at stated intervals, the proceeds of which go to the Home. Of course the societies get their money's worth in return; for they have, we may be sure, more than enough applicants for the nominations their donation entitles them to, but the *fête* thus organised shows that the working men of Ipswich are alive to the importance of helping themselves. The Home is managed on the same healthy principle. There are no idlers there, and anyone who has experienced the *ennui* of convalescence will know how powerfully this rule will operate in hastening recovery. Every inmate, except in cases of urgent need, pays five shillings a week, and the donor's or subscriber's nomination holds good for three weeks, no patient remaining for a longer period than that of two nominations, unless by the special leave of the committee. In addition to these, a limited number of paying patients are admitted, the charge for them being 15s. per week. In all cases, however, the inmates, when practicable, must take an active part in the work of the Home. The women do the mending, and the men polish the boots, and other work of that sort, besides which every patient is supposed to make his or her bed and leave the room "tidy" in the morning. This, however, leaves plenty of time for reading, conversation, smoking, and, above all, walking, for the patients are encouraged to take as much exercise as is good for their individual cases. Very few, either of the men or the women, who were at the Home on the occasion of my visit seemed to ail very much, except one or two of the fresh arrivals, who—the men, I mean—were recovering from accidents. From what has already been said, it will be understood that the inmates mostly belong to the class of small tradesmen, mechanics, artisans, and servants, and this year over four hundred men and women will have passed through the Home. The advantages which they derive are, it is needless to say, by no means recompensed for by the almost nominal payments they make; but the very fact of the payment, coupled with the interest they all take in the daily life of the Home, gives to the place an air of cheerfulness, contentment, and independence which is wanting in some more pretentious institutions. Of course there are complaints sometimes—even the most carefully regulated establishments go wrong occasionally; but it is a curious fact that the better the class of inmates, the more superior their education, the higher the standard of living to which they have been accustomed, the more seldom do they grumble. It is the upstarts, the "beggars on horseback" for the time being, who give themselves airs, and as they wax fat and healthy show signs of kicking, and complain without a cause. That is the experience of us all in larger worlds than this little one in Suffolk, and will continue to be the experience until human nature has been revolutionised. I ought to add that the Mercers' Company have been good friends to this Felixstowe Home, having given regularly two hundred guineas for the last five years, and that though the new building is now free of debt, there is no lack of good uses to which the further contributions of the charitable may be put.

